# Aging of Pelvic Floor in Animal Models: A Sistematic Review of Literature on the Role of the Extracellular Matrix in the Development of Pelvic Floor Prolapse

**DOI:** 10.3389/fmed.2022.863945

**Published:** 2022-04-15

**Authors:** Barbara Gardella, Annachiara Licia Scatigno, Giacomo Belli, Andrea Gritti, Silvia Damiana Visoná, Mattia Dominoni

**Affiliations:** ^1^Department of Obstetrics and Gynecology, Fondazione IRCCS Policlinico San Matteo, University of Pavia, Pavia, Italy; ^2^Department of Public Health, Experimental and Forensic Medicine, University of Pavia, Pavia, Italy

**Keywords:** pelvic floor disfunction, animal models, pelvic organ prolapse, aging, connective tissue

## Abstract

Pelvic organ prolapse (POP) affects many women and contributes significantly to a decrease in their quality of life causing urinary and/or fecal incontinence, sexual dysfunction and dyspareunia. To better understand POP pathophysiology, prevention and treatment, many researchers resorted to evaluating animal models. Regarding this example and because POP affects principally older women, our aim was to provide an overview of literature on the possible biomechanical changes that occur in the vaginas of animal models and their supportive structures as a consequence of aging. Papers published online from 2000 until May 2021 were considered and particular attention was given to articles reporting the effects of aging on the microscopic structure of the vagina and pelvic ligaments in animal models. Most research has been conducted on rodents because their vagina structure is well characterized and similar to those of humans; furthermore, they are cost effective. The main findings concern protein structures of the connective tissue, known as elastin and collagen. We have noticed a significant discordance regarding the quantitative changes in elastin and collagen related to aging, especially because it is difficult to detect them in animal specimens. However, it seems to be clear that aging affects the qualitative properties of elastin and collagen leading to aberrant forms which may affect the elasticity and the resilience of tissues leading to pelvic floor disease. The analysis of histological changes of pelvic floor tissues related to aging underlines how these topics appear to be not fully understood so far and that more research is necessary.

## Introduction

The female pelvis is a dynamic structure, responsible for pelvic organ support. This support is provided directly by the vagina and indirectly by the structures involved in vaginal support, a complex tree-levels system composed of striated muscle, smooth muscle and connective tissue, as previously described by De Lancey (1).

Pelvic organ prolapse (POP) is defined as a downward displacement of pelvic organs, resulting in herniation of those organs into or through the vagina. It could involve the anterior compartment (cystocele or bladder prolapse), the uterus and/or the posterior compartment (which may be a rectocele and/or an enterocele) (2).

It is a disturbing problem which affects many women and contributes significantly to a decrease in their quality of life (3), but there is not consensus about the true prevalence of POP: indeed, some degree of prolapse is present in 41 to 50% of women on physical examination (3), but only 3% of patients report symptoms (4).

Pelvic organ prolapse may be associated with a wide range of symptoms: a vaginal lump, bulge or a “dragging” sensation, sexual dysfunction, dyspareunia, urinary and/or fecal incontinence, voiding dysfunction or obstructed defecation.

In addition, this disorder has an emotional impact resulting in psychosocial distress, isolation, anxiety and depression (5).

The pathophysiology of POP is still not well understood, but it is known that several risk factors have been associated with it, such as genetic predisposition, aberrant connective tissue metabolism, pregnancies, vaginal delivery, hormonal levels and menopause, obesity, aging, previous hysterectomy and constipation (6).

To better understand the pathophysiology, prevention and treatment of POP, a large number of researchers resorted to evaluating animal models, as an example of their use in other clinical fields such as general surgery for transplantation, or trials about pain perception, or would reparation and tissue regeneration (7–9).

Animal models are convenient for several reasons: they avoid ethical questions surrounding human studies, they reduce the long time span prior to patients becoming symptomatic, allow the opportunity to test hypotheses in a controlled environment without interfering co-factors.

These models can mimic different human histological, anatomical or hormonal characteristics, but finding an optimal model is challenging because no one model represents all of these characteristics at the same time and models have several differences with humans: to name a few, animals are quadrupeds and have a different pelvic floor structure and a different birth process. Since POP is a disorder that mainly affects postmenopausal and older women, we focused on the possible effects of vaginal and pelvic floor aging starting from a perspective of analyzing animal models, particularly on the biomechanical changes that occur within their vaginas and their supportive structures as a consequence of aging and hormonal changes, which perhaps favor the onset of these pelvic floor disorders in humans as well.

## Materials and Methods

In order to achieve a review of literature regarding the histological modifications of uterine tissue induced by aging in animal models, the most significant medical databases, including PubMed, Cochrane Database of Systematic Reviews, EMBASE, and Web of Science, were consulted, according to a combination of the following keywords: “pelvic floor animal model”, “pelvic organ prolapse animal model,” “aging and pelvic floor” including pluralization and US English/United Kingdom English spelling variations and suffixes/prefixes.

**FIGURE 1:**
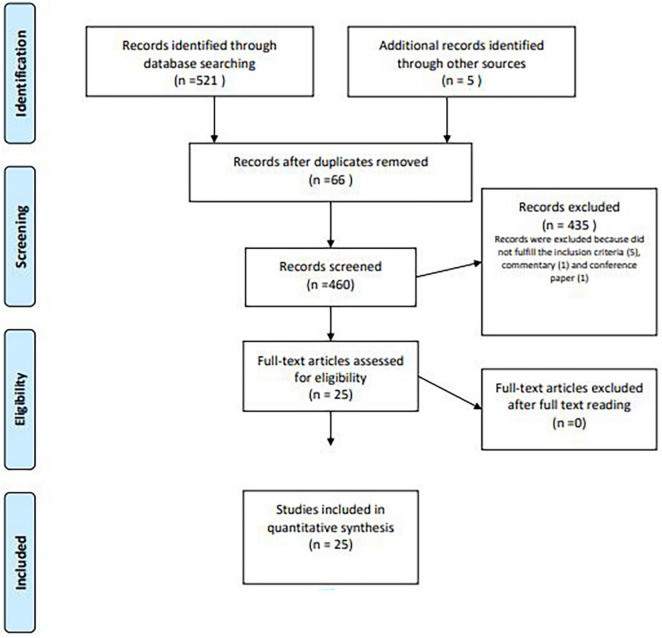
PRISMA flow diagram of study selection process.

For our analysis, all papers published online from 2000 until May 2021, including literature reviews, case series, and retrospective or prospective trials, were considered. We performed a systematic search using the Preferred Reporting Items for Systematic Reviews and Meta-Analyses (PRISMA) literature selection process (Figure 1).

Two authors independently (S.A.L. and B.G.) searched reference lists of recognized manuscripts in order to integrate the literature into the review.

Eligible studies were those in which POP was studied in animal models; particular attention was given to articles reporting the effects on the microscopical structure of the vagina and pelvic ligaments.

Papers have been included in the analysis if the following criteria were met: (I) histological characteristics of uterine tissue modification induced by aging, (II) differences of uterine modification among animal models, (III) functional modifications of uterine tissue induced by aging.

The selected articles were assessed as full-text literature and the resulting information was tabulated.

Exclusion criteria were as follows: (I) case reports were excluded in the present literature review when considered less significant in this field; (II) abstracts of medical conferences, editorials, and preliminary studies in non-animal models; (III) multimedia materials regarding the objective of the study; and (IV) papers written in languages other than English.

For each selected study, two investigators (S.A.L. and B.G.) independently assessed the risk of bias to ensure validity and overcome eventual selection, performance, detection, attrition, and reporting bias, according to the Cochrane Handbook for Systematic Reviews of Interventions (10–12).

Bias across studies as well as bias and risks related to the source of funding and conflict of interest of authors of the included studies, were assessed. Eventual disagreements were resolved through discussion.

The data extraction form was validated by the researchers S.A.L. and B.G. and independently extracted by the two researchers.

It was not possible to conduct a meta-analysis because of the heterogeneity of the included study designs and outcome measures.

Regarding the histological analysis methods proposed by the articles collected in this review, there have been many in addition to hematoxylin and eosin staining (H&Es) that were present basically in all of the papers related to the histological findings, which are after all the most commonly used staining methods in pathology.

Masson’s trichrome stain has been widely used as well, since it is tailored to characterize cells from connective tissues. In this combination of stains, the methyl blue is used to better detect collagen and elastic fibers (13).

Fundamental to the study of elastic fibers are the Van Gieson Verhoff stain (VGVs) and the Hart’s stain. These methods are basically based on a variety of ionic reagent that bonds with elastin, getting a better possible evaluation of connective tissue components. Using VGVs, elastin looks black while collagen is colored pink and in Hart’s stain elastin appears purple-black (14, 15).

Some studies reported, as well, Immunofluorescence (IF) and Immunohistochemistry (IHC), which are immunology-based method, related to the antigen-antibody binding, allow the identification of tissue-specific epitopes. In these works, IF and IHC were tailored to detect extracellular matrix components (14, 16). In the IF technique, fluorophores are used to mark the expression of the correct antigen-antibody binding and the samples should be observed under a fluorescent microscope or a confocal microscope. In IHC, a brown-colored precipitate (3,3′-diaminobenzidine) is used to detect the expression of the antigen studied and this precipitate forms where the antibodies have bound to the known antigen.

## Results

For our purpose, a total of 521 studies were identified by the search strategy and 5 were identified through the references.

Duplicated papers, presented in more than one database, and irrelevant works were not considered for our analysis; furthermore, after the removal of articles not published in English or published before 2000, 460 articles were screened by title and/or abstract.

At the end, 25 studies were included and compared, focusing on the possible biomechanical and microscopical changes in the vagina and in the pelvic floor induced by aging in animal models (Figure 1).

It was difficult to detect the histological changes of elastic fibers related to aging in animal specimen, basically because animals have a limited life span compared to humans (17).

Some studies performed to mimic elastic fibers deficiencies linked to aging have been reported in literature, essentially based on an experimentally induced degradation of elastic fibers.

The main findings concern protein structures of the connective tissue, known as elastin and collagen.

The main methods of histological analysis detected in this review have been:

• H&E and Masson’s stain, where it was possible only to observe the presence of connective tissue without getting information about their components (18);
• Van Gieson Verhoff and Hart’s stain that can lead to preliminary differentiation between elastin and collagen (in VGVs elastin gets a black staining while collagen is colored pink);
• Finally, few articles used immunohistochemistry, with a detailed histological analysis of collagen components (14, 15).

In Table 1 we reported the most significant articles about the link between aging and the changes in the connective tissue components of the pelvic floor. In Table 2 we reported all of the studies included in the review.

**TABLE 1:** Principal findings about the link between aging and histological changes in animal models pelvic floor.

References	Type of animal model	Number of animal samples	Staining methods	Results
Chen et al. (13)	Humans and rats	60 rats divided in 3 groups	Immunohistochemistry (anti-RAGE; anti-AGE; anti-collagen I)	Lower expression of collagen I in elderly; No difference of AGE and RAGE in different age
Mao et al. (25)	Rats	36 rats divided in 4 groups	Masson’s Trichrome stain; Picro-Sirius Red Staining; Immunohistochemistry (anti- a-SMA)	Lower expression of collagen III in elderly; Higher expression of collagen I in elderly
Shahryarinejad et al. (26)	Humans and macaques	Review article	Masson’s Trichrome stain; Verhoff-Van Gieson stain	Lower expression of collagen III in elderly; No alteration of collagen I expression in elderly
Ferreira et al. (15)	Mice	240 samples divided in 4 groups	Immunofluorescence (anti- Col1A1; anti-elastin)	Lower expression of elastin and collagen in elderly
Mori Da Cunha et al. (18)	Rodents, lagomorphs, sheep, non-humans primates	Review article	Review	Downregulation of elastin metabolic genes
Rahn et al. (22)	Mice	53 Fbln3−/− animals 74 Fbln5−/− animals	H&E; Hart’s stain	Elastin seems to be more tortuous, porous and frayed in elderly
Couri et al. (23)	Mice	Review article	Masson’s Trichrome stain; Verhoff-Van Gieson stain	No alteration of elastin expression in elderly

**TABLE 2:** Studies included in the systematic review.

References	Type of article	Type of animal model	Conclusions and results
Urbankova et al. (12)	Original article	Sheep	In sheep, menopause, so aging, affect the biomechanical properties of the vagina: it increase the stiffness of the mid-vagina.
Chen et al. (13)	Research article	Human and rats	Lower expression of collagen I in elderly; No difference of AGE and RAGE in different
Couri et al. (14)	Research article	Mice	Loxl1 knockout mice demonstrate pathology primarily characterized by enlargement of the vagina.
Ferreira et al. (15)	Research article	Mice	Lower expression of elastin and collagen in elderly
Chin et al. (16)	Research article	Mice	Vaginal fibulin-5 during development is crucial for baseline pelvic organ support and is also important for protection and recovery from parturition- and elastase-induced prolapse
Mori da Cunha et al. (18)	Review article	Rodents, swine, rabbit, sheep and NHPs	In rodents, aging led to a reduction of estradiol and to a downregulation of the Lox3 and Lox4 expression (which are involved in the elastin metabolism) and seems to have an effect on the healing process of the vagina. In baboons, aging did not coincide with more signs of POP.
Jiang et al. (19)	Original article	Mice	Aging is a high-risk factor for pelvic floor disorders. The failure of elastic fiber synthesis and assembly due to the decline in expression levels of elastin and LOX family members during aging may explain the molecular mechanism causing pelvic floor disorders.
Alperin et al. (20)	Original article	Mice	LOXL1 mutation results in a global defect in connective tissues and correlates with altered biomechanical behavior of the vagina and supportive tissues.
Drewes et al. (21)	Research article	mice	Synthesis and assembly of elastic fibers are crucial for recovery of pelvic organ support after vaginal delivery and that disordered elastic fiber homeostasis is a primary event in the pathogenesis of pelvic organ prolapse in mice in adulthood
Budatha et al. (22)	Research article	Mice	POP is an acquired disorder of extracellular matrix during old age and that therapies targeting matrix proteases may be successful for preventing or ameliorating POP
Couri et al. (23)	Review article	Rodents, swine, rabbit, sheep and NHPs	No alteration of elastin expression in elderly
Rahn et al. (24)	Original article	Mice	Elastin seem to be more tortuous, porous and frayed in elderly
Mao et al. (25)	Research article	Rats	Lower expression of collagen III in elderly; Higher expression of collagen I in elderly
Shahryarinejad et al. (26)	Review article	Humans and macaques	Lower expression of collagen III in elderly; No alteration of collagen I expression in elderly
Lee et al. (31)	Original article	Mice	Elastin disorganization that occurs in old age may lead to functional abnormalities
McLaughlin et al. (32)	Original article	Mice	Efemp1(−/−) mice exhibited reduced reproductivity, and displayed an early onset of aging-associated phenotypes including reduced lifespan, decreased body mass, POP, lordokyphosis, reduced hair growth, and generalized fat, muscle and organ atrophy
Abramowitch et al. (33)	Review article	Rodents, swine, rabbit, sheep and NHPs	Aging affects the biomechanical properties of animal and human tissues
Jackson et al. (34)	Research article	Ewes	Risk factors for POP in ewes are reproductive rates, age, gain in weight
Mattson et al. (40)	Residents’ papers	Baboon	In baboons, aging did not coincide with more signs of POP
Rizk et al. (49)	Comparative study	Rat	All biomarkers of urogenital aging studied were significantly increased in old compared to young-adult sham rats. Ovariectomy significantly increased these changes further in old versus young-adult rats with either smaller or larger differential effect than aging compared to young-adult sham animals. Ovariectomy significantly exacerbates normative urogenital aging changes in rats.
Rizk et al. (50)	Research article	Rat	Estrogen and/or ghrelin significantly increased or decreased, respectively, circulating growth hormone in old and young adult rats. Estrogen/ghrelin administration reversed pelvic floor muscle aging changes in old ovariectomized rats through growth hormone production.
Shveiky et al. (51)	Original article	Rat	In this rat model, age impaired vaginal wound healing, which was reflected in the altered inflammatory response to injury and reduced tissue strength
Moalli et al. (52)	Original article	Rat	Ovariectomy has a differential effect on the tissues of young vs. middle aged rats.
Shveiky et al. (53)	Research article	rat	The presence of the inflammatory cytokine macrophage-migration inhibitory factor (MIF) in the aged rats suggested wound healing was impaired
Menachem-Zidon et al. (54)	Original article	Rat	Excessive and prolonged macrophage response in older rats may contribute to poor wound healing in the vagina

A significant disagreement has been identified in the literature concerning elastin changes related to aging.

Some authors observed a downregulation of genes implicated in elastin metabolism (19). Jiang et al., indeed, explored the expression of elastin and lysyl oxidase (LOX), proteins related to elastin metabolism, among the reproductive system of natural aging mice, detecting a significant decline in the expression of elastin and LOX proteins (20).

Also, Alperin in 2008 agreed that mice deficient in LOXL1 develop pelvic organ prolapse because they present mechanically weaker tissue: LOXL1 mutation results in a global defect in connective tissue and correlates with altered biomechanical behavior of the vagina and its supportive tissues (21).

Instead, fibulin5 knock out (Flbn5 KO) mice develop prolapse early in life and the majority of animals will develop POP even if nulliparous (22). A conserved motif on the Fbln5 gene regulates the expression of matrix metalloproteases (MMPs) (23). Interestingly, when this motif is disrupted, mice display altered MMP activity, suggesting that the Fbln5 gene regulates pelvic connective tissues both by regulation of elastic fiber organization and by MMP inhibition (23). But mice with a deficiency in both Fbln5 and Mmp9 (Fbln5 and Mmp9 double KO) have significantly reduced POP development, indicating that deficiency in Mmp9 could possibly prevent POP in up to 60% of Fbln5 KO mice.

Nevertheless, other authors do not detect any differences in the amount of elastin in aging, reporting that the elastic fiber turnover seems to be constant in female reproductive organs (24). Instead in this context, the significant change is that the elastin seems to be more tortuous, porous and frayed (25).

Referring to collagen, the main extracellular components of the genitourinary system are collagen I and III (14): collagen I offers strength and rigidity to the tissue, while collagen III is more malleable and flexible (26). Some authors observe that in elderly women it seems that the expression of collagen I increases, while the amount of collagen III decreases (26, 27). On the other hand, other authors, detect that the expression of collagen I was lower in elderly women (14, 16). Chen et al., investigated changes in advanced glycation end-products (AGEs) and their receptor (RAGE) by an immunohistochemical approach, since the AGE-RAGE pathway is involved in dysregulated collagen metabolism. However, differently from collagen I, no difference in AGE or RAGE expression have been detected in different ages (14).

## Discussion

Among animal models, rodents (both mice and rats) are the most widely used species for POP studies, especially when evaluating connective tissue support (28) because their vaginal structure is well characterized and similar to those of humans, as well as the anatomy and histological architecture of the uterosacral, cardinal and round ligaments (29, 30).

Rodents are cost-effective and easy to work with a large number; due to the length of their estrus cycles and of their gestations, both mice and rats allow a more rapid evaluation of processes that would normally take a longer period of time compared to other species.

Furthermore, transgenic knockout mouse models, in particular the lysis oxidase like-1 (LOXL-1) deficient mice, display a POP phenotype very similar to the human clinical condition (31, 32): it has been demonstrated that LOXL1-deficient mice primarily prolapse after delivery and that parity increases the rate of prolapse in these models (31). Also, mice deficient in fibulin 5 (FBLN5) develop prolapse as early as 12 weeks of age, even if nulliparous (21).

Rabbits have many of the same advantages of rats and they are also larger (which means larger tissue acquisition and easy access to the vagina transperineally). However, the anatomy of the rabbit vagina is completely different from humans, so they are rarely used (28).

The pelvic floor in sheep is surprisingly similar to the woman’s one and may become the next appropriate alternative to the more expensive and less accessible primate model (33); uterovaginal prolapse occurs commonly in sheep, sometimes spontaneously (whose etiology is not well understood) (34, 35), but in most cases it is due to predisposing factors, especially pregnancy and vaginal birth (36). In addition, they are inexpensive, available in large numbers and are an established reproductive model for humans (33) because they have prolonged labors, relatively large fetuses and frequent dystocia (37).

Finally, non-human primates (NHPs) are considered the experimental model for pelvic organ prolapse most similar to humans (27, 38, 39), even because, differently from other animal models, NHPs also have levator ani muscles with analogous functions to the iliococcygeus, pubococcygeus, and puborectalis muscles in humans (33). The disadvantages of NHPs as animal model include the long pregnancy and the long time to develop spontaneous POP, the costs, the level of expertise needed to handle them and ethical constraints (18), so for these reasons they are less common used. In particular, baboons have similar anatomy to women and squirrel monkeys and could be useful to assess surgical procedures (40).

Anatomically, one of the main differences between humans and every other animal model is that humans are bipedal. Indeed, as a consequence of being bipedal, humans have lost the need for a tail and have recruited the levator ani, which is important for tail function in many animals, to counter abdominal pressure and gravity (27).

On the other hand, many mammals appear to have a similar connective tissue support as in humans.

In women, the connective tissue contains collagen, elastic fibers, proteoglycans, an extracellular matrix and a significant amount of smooth muscle. In the vagina, collagen is the primary structural component (84%) with an intermediate amount of elastin (13%).

Basically, the content of collagen I is the primary determinant of tensile strength (41), while collagens III and V help to limit the diameters of collagen fibrils, so that an increase in collagens III and V is associated with decreases in the mechanical integrity of connective tissue (42).

As we all know, the remodeling of the connective tissue affects the mechanical integrity of the vagina and its supportive tissue (43). Collagen and elastin degrading enzymes like matrix metalloproteinases (MMP) seem to be upregulated in the vagina of women with prolapse (44–46) and mice with null mutations in genes involved in elastin synthesis develop spontaneous prolapse (31, 46).

Most research about POP in animal models focused on what the possible risk factors are, which impact the remodeling of connective tissue.

The role of extracellular matrix in pelvic tissue structures was evaluated also during pregnancy in animal models; these results underline as the pregnancy and delivery play a significant role in the modification of pelvic structures. During pregnancy, Alperin et al. reported an increase in collagen V pelvic muscles (coccygeus and puborectalis) in late-pregnant rats. In addition, the authors observed a decrease of enzymatic crosslinks decreased in these structures, despite an increase of glycosylated crosslinks (47). Similarly, pregnancy and delivery in laboratoristic rats may cause the dissociation of collagen fiber due to the changes in the density and thickness of the collagen structure. In addition in uterus there was reported a diminution of elastin of extracellular matrix, caused by the remodellation of pelvic structures in order to balance the pregnancy modification of ligament and connectives (47). These data supported the hypothesis that pregnancy and delivery play a possible role in the modification of extracellular matrix in pre-menopause with a plausible reflection in the aging of pelvic strtuctures.

Interestingly, hormones play a key role in regulating the mechanical properties of the supportive connective tissues, in particular the changes that occur during premenopausal and menopausal periods (48) and persist during old age. As demonstrated by Rizk et al. (49), ovariectomy significantly exacerbates urogenital aging changes in rats. Furthermore, in old rats estrogen/ghrelin administration reversed pelvic floor aging changes in ovariectomized rats (50).

As shown above, most of the studies are aimed to detect the amount of elastin in the female reproductive system. In this context, any difference in the quantity of elastin, related to aging, has been reported and this has to do with the elastic fiber turnover, which seems to be constant in the female reproductive system (23, 24). What is changing in elderly women seems to be not so much the amount of elastin, but the quality of elastin: indeed, elastin appears more tortuous, frayed and porous in elderly (24). Although there are not tailored studies about the effect of these qualitative changes of the elastin on the female reproductive system, it is possible to assume that alteration of elastogenesis, leading to aberrant forms of elastin, may affect the elasticity and resilience of elastin fibers, predisposing women to pelvic floor disease, such as POP.

Talking about collagen, collagen I expression in the female reproductive system seem to be lower in elderly subjects (14). These findings lead to the consideration that, at the same time, the percentage of collagen III in these tissues, which is define as malleable and flexible, arises in elderly, proportionally to the lower expression of collagen I. This “shift” in collagen proportions in pelvic tissues in elderly can help to explain the decrease of pelvic floor biomechanical strength and properties related to aging. However, different studies reported discordant findings about collagen I expression in elderly, in the genital-urinary female system (27, 28), probably because of different targets of study or different methods or standards.

The analysis of histological changes of the pelvic floor tissues related to aging underline how these topics appear to be not fully understood so far, and how sometimes papers referring to them seem controversial. These assessments are even more complex and controversial if applied to the role of the aging regarding the pathophysiology of POP.

In mice, it seems that both accelerated and naturally aging led to a reduction of estradiol, respectively of 62 and 44%, suggesting a connection between advancing age and hormonal changes (19). This was also observed in aging rats (51, 52).

Furthermore, aging seems to be associated with the downregulation of gene and protein expression of Lox3 and Lox4, which play a role in the synthesis of elastic fibers (19).

Finally, aging seems to have an effect on the healing process of the vagina. Indeed, the presence of the inflammatory cytokine macrophage-migration inhibitory factor (MIF) in the aged rats suggested wound healing was impaired (53). While, 30 days after injury, old rats regain only 15% of its original strength and compliance whereas young rats recovered for 60%. This was associated with a delayed and long-lasting expression of MIF (macrophage response) (52, 54).

Instead in baboons, aging did not coincide with more signs of POP (40).

## Conclusion

We recognize that no animal model for studying POP is perfect, but rodents are a preferred model when evaluating connective tissue support because their vaginal structure is well characterized and similar to those in humans. Furthermore, they are cost effective, easy to work with a large number and allow researchers to work with transgenic knockout species.

Through the study of the literature, it’s clear that the remodeling of the connective tissue affects the mechanical integrity of the vagina and its supportive tissue. However, the disagreement among the authors about elastin and collagen quantitative changes related to aging reflects the difficulty to detect how much are the histological changes of elastic fibers in animal specimens. Maybe this is due to two of the principal limitations in the use of animal models in clinical research: indeed, animals have a limited life span compared to humans; moreover, small animals are frequently used.

Instead, as seen in almost all of the selected studies, there seems to be agreement that aging affects elastin and collagen properties leading to aberrant forms which may affect the elasticity and resilience of the tissue, predisposing women to pelvic floor disease.

About clinical implication, we could say that these researches are fundamental for the development of new types of local therapies: for example, therapies targeting matrix proteases may be successful for preventing or ameliorating POP.

Thus, according to us, more research is also necessary in humans to understand if aging is involved in the onset of pelvic floor disorder as in animals models.

## Data Availability Statement

The original contributions presented in the study are included in the article/supplementary material, further inquiries can be directed to the corresponding author.

## Author Contributions

AS and GB wrote the manuscript. BG and MD designed the structure of the manuscript. AS, GB, and AG contributed to the literature search. MD, BG, and SV reviewed and revised the initial manuscript and approved the final manuscript as submitted. All authors contributed to the article and approved the submitted version.

## Conflict of Interest

The authors declare that the research was conducted in the absence of any commercial or financial relationships that could be construed as a potential conflict of interest.

## Publisher’s Note

All claims expressed in this article are solely those of the authors and do not necessarily represent those of their affiliated organizations, or those of the publisher, the editors and the reviewers. Any product that may be evaluated in this article, or claim that may be made by its manufacturer, is not guaranteed or endorsed by the publisher.
